# Developmental Defect of Enamel in Permanent Teeth Associated With Chronic Endodontic Abscess in Deciduous Teeth: A Retrospective Study

**DOI:** 10.1002/cre2.70185

**Published:** 2025-07-23

**Authors:** Federica Guglielmi, Angela Malatesta, Anna Alessandri‐Bonetti, Patrizia Gallenzi

**Affiliations:** ^1^ Institute of Dental Clinic, A. Gemelli University Policlinic IRCCS Catholic University of the Sacred Heart Rome Italy; ^2^ Postgraduate School of Orthodontics Catholic University of the Sacred Heart Rome Italy; ^3^ Postgraduate School of Paediatric Dentistry Catholic University of the Sacred Heart Rome Italy

**Keywords:** abnormalities, dental pulp necrosis, pediatric dentistry, tooth injuries

## Abstract

**Objectives:**

Destructive carious lesions on deciduous teeth often result in dental abscesses. Sometimes, the exudative process may extend to the dental follicle of the permanent tooth, leading to various types of consequences. This study primarily seeks to determine the prevalence of developmental defects of enamel (DDE) in premolars whose predecessors developed endodontic abscesses. Furthermore, it investigates how the prevalence of DDE is influenced by the type of treatment the affected deciduous molar received. Lastly, the study compares the prevalence of DDE between maxillary and mandibular premolars.

**Material and Methods:**

Demographics, medical and dental history, and records of DDE were extracted from the medical records of 1164 pediatric patients. DDE of 107 premolars from patients who had experienced abscesses in their deciduous molars were compared to DDE of 107 premolars from patients who naturally shed healthy deciduous molars. DDE were also compared between different treatment modalities and anatomical regions. Fisher's exact tests were used to compare groups, while demographic data were analyzed by descriptive statistics and reported as mean ± standard deviation or as median and interquartile range for the continuous variables.

**Results:**

Compared to premolars whose predecessors did not exhibit signs of pathology, those that developed endodontic abscesses reported a higher prevalence of DDE (57% vs. 17.8%; OR 6.14; *p* < 0.0001). Endodontic treatment on deciduous molars was associated with higher DDE prevalence compared to surgical treatment (70.2% vs. 46.7%; OR 2.69; *p* = 0.016). Maxillary premolars showed a higher prevalence of DDE compared to mandibular premolars (75.4% vs. 24.6%; OR 5.23; *p* = 0.00008).

**Conclusions:**

Chronic endodontic abscess on deciduous molars significantly increases the risk of DDE in the corresponding premolars. ET on deciduous molars is associated with higher incidence of DDE compared to extraction. Maxillary premolars are more likely to develop DDE than mandibular premolars.

## 1 Introduction

Developmental defects of enamel (DDE) are a common defect of the dental enamel, which may present with a prevalence ranging up to 50% of the population (Dummer et al. 1990). DDE occur due to a variety of interacting factors ranging from genetic abnormalities to environmental insults (Seow 2014). Clinically, DDE often presents with problems like discoloration and esthetics (Seow 2014) but also tooth sensitivity, and higher odds of developing dental caries (Costa et al. 2017); therefore, understanding factors associated with DDE development is an important matter.

Several factors have been identified to be associated with DDE in children including preterm birth and vitamin and calcium deficiency (Bensi et al. 2020; Neto et al. 2020); however, little is known regarding factors influencing DDE in permanent dentition. Few studies suggested that dental caries on deciduous teeth increases the risk of developing demarcated opacities on permanent successors (Broadbent et al. 2005; Lo et al. 2003), and a recent narrative review reported abnormalities on deciduous teeth including ankylosis, traumatic injuries, and mutilation to be correlated to DDE (Collignon et al. 2022). The mechanisms involved in DDE development were suggested to be due to the spread of inflammation to the underlying permanent tooth germ (Collignon et al. 2022). Several studies reported that suppuration resulting from deciduous tooth necrosis can spread apically through the follicular sac, potentially causing damage to the developing permanent tooth, leading to enamel defects or even stopping tooth formation (Bauer 1946; Valderhaug 1974).

Due to the significant impact of DDE on the overall patient's dental health, the primary aim of this study was to determine if chronic endodontic abscess in deciduous teeth is associated with DDE in permanent successors.

Our hypothesis was that premolars whose predecessors developed endodontic abscesses would present a higher risk of DDE compared to premolars whose predecessors did not present any inflammatory process. Our secondary aims were to compare differences in DDE according to the type of treatment received by the deciduous teeth and their anatomical location. Specifically, we compared endodontic treatment to surgical treatment, and maxillary to mandibular premolars. Because these aims were exploratory, we did not have any a priori hypothesis.

## 2 Materials and Methods

### 2.1 Study Design and Eligibility Criteria

This retrospective observational study was conducted on the medical records of patients aged 6–17 years treated from January 2018 to December 2023. Subjects who had a prior history of endodontic abscess on at least one deciduous molar and which had developed on corresponding permanent premolars were selected as the study group (SG). After selecting patients with endodontic abscess history, a control group (CG) of patients who had naturally shed healthy deciduous molars without any inflammatory process on the deciduous teeth (i.e., dental caries), was selected and matched with the SG by sex and age.

Patients with a story of dental trauma or orthodontic treatment, those diagnosed with dental fluorosis, molar incisor hypomineralization (MIH), or amelogenesis imperfecta were excluded from the study. Additionally, patients who did not provide informed consent were not included.

### 2.2 Data Collection

The extracted data consisted of records including histories and dedicated questionnaires, which allowed for the assessment of exposure to the risk factor “dental abscess,” and for information regarding the presence of DDE. The enamel of erupted premolars had been assessed for each patient using the DDE modified index, which classifies enamel development defects into categories as “demarcated opacities,” “diffuse opacities,” “hypoplasia,” “other types of defects and their combinations” with the corresponding subtypes (Clarkson 1989). The DDE assessment was performed by experienced pediatric dentists at the Paediatric Dentistry Unit of the A. Gemelli Polyclinic. While a formal calibration exercise specifically for this study was not conducted due to its retrospective nature, the DDE modified index was consistently applied by the clinical team in their routine practice. Data extraction for the study was performed independently by researchers not directly involved in the original clinical diagnosis to minimize bias.

DDE presence was compared between SG and CG. Then, the SG was further analyzed depending on the treatment deciduous teeth underwent: DDE presence was compared between premolars whose deciduous molars with abscess were extracted (E) and premolars whose deciduous molars underwent endodontic therapy (ET).

### 2.3 Type of Treatments for Deciduous Molars With Endodontic Abscess

All treatments of the SG were conducted by an experienced and qualified pediatric dentist and were performed under local anesthesia without any sedation technique or general anesthesia. The clinical protocol for endodontically treated deciduous teeth included several steps. First, a topical anesthetic solution was applied to a dry mucosa for 2 min (Lidocaina Spray 15%, Ogna), followed by local anesthesia (3% mepivacaine with epinephrine 1:100,000) and isolation of the operative field with a rubber dam. Access to the pulp chamber was achieved using a round diamond bur and an Endo Z bur. Working length was determined with an apex locator (Root ZX Morita), and the canals were instrumented with ProTaper Next files (Maillefer) under copious irrigation with 5.25% NaOCl. Finally, a dressing of an association of calcium hydroxide and iodoform paste (Caliform OGNA) was applied, providing acid‐neutralizing action and stimulation of repair processes. An occlusal intermediate dressing of zinc oxide (Cavit 3M) was placed, and after 20 days, the occlusal dressing was replaced with a definitive composite resin filling (Parameswaran 2023; Wang et al. 2024; Tewari et al. 2022).

When the extraction was indicated, a radiographic examination was performed first. Following the administration of topical anesthesia (Lidocaine Spray 15%, Ogna) and local anesthesia (mepivacaine 3% with epinephrine 1:100,000), the extraction procedure was performed. The clinician used pediatric forceps and an elevator, as indicated in the pediatric oral surgery protocols (American Academy of Pediatric Dentistry 2023).

### 2.4 Sample Size Calculation

As suggested by Heloisa Clara Santos Sousa et al. (2020), a formula for comparing groups according to qualitative variables in split samples was used to calculate the minimum sample size, considering a 95% confidence interval, normal curve point for error 1.96 (5%) and error 0.84 (20%). An average percentage of 31.4% enamel defects in premolars whose predecessors had undergone pulpal therapy and 50% for those extracted for pulpal necrosis and without therapy was adopted. A minimum sample size of 105 premolars per group was obtained (Sousa et al. 2020).

### 2.5 Statistical Analysis

Demographic data were summarized using descriptive statistics and presented as mean ± standard deviation, or median and interquartile range for continuous variables, depending on the normality of distribution as determined by the Shapiro–Wilk test. Categorical variables were reported as absolute frequencies and percentages (Shapiro and Wilk 1965). The association between independent variables on the prevalence of DDE in the overall sample and in each of the groups separately was compared using Fisher's exact tests (alpha = 0.05). The odds ratio (OR) and 95% confidence interval were also calculated. Data extracted from medical records were organized and analyzed using Microsoft Excel v.2021.

## 3 Results

Out of the 1164 charts of consecutive patients, 107 premolars from 47 patients who had experienced endodontic abscess and/or fistula in deciduous molars were selected for the SG and compared to 107 premolars from 47 patients in the CG; resulting in a final sample of 214 premolars from 94 patients. Participants' mean age was 13.07 ± 3.7 years; 48.6% males and 51.4% females. DDE was observed in 61/107 (57%) of the premolars in the SG, compared to only 19/107 (17.8%) of the premolars in the CG. The difference was found to be statistically significant with an odds ratio (OR) of 6.14 (95% CI 3.28–11.49; *p* < 0.0001). The most common DDE observed in the SG was diffuse patchy, while demarcated opacities white/cream and diffuse‐confluent defects were the most common DDE observed in the CG.

**Figure 1:**
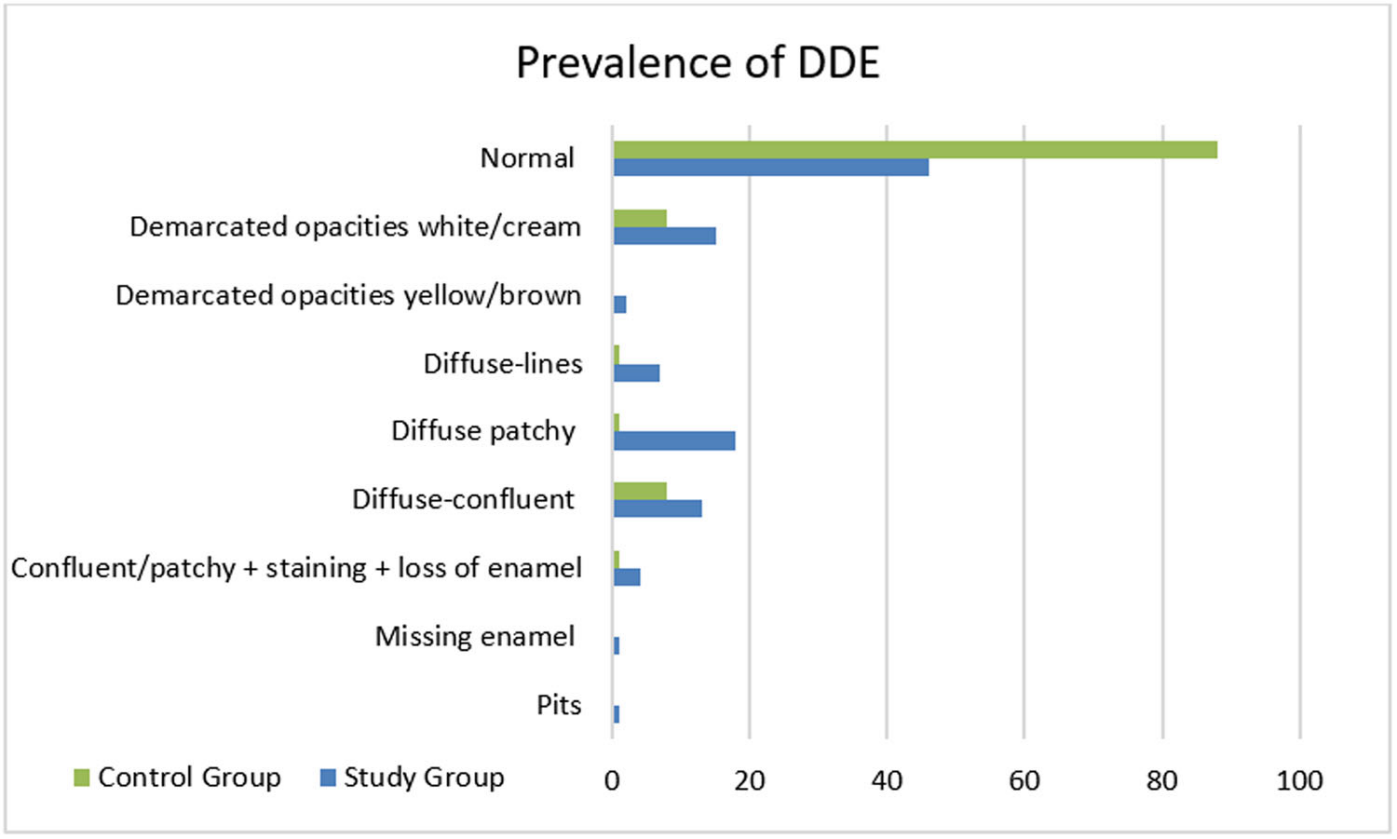
Distribution of types of DDE observed on premolars in the SG and CG.

Table 1 and Figure 1 show the distribution of prevalence and types of DDE observed on premolars in the SG and CG.

**Table 1:** distribution of prevalence and types of DDE observed on premolars in the SG and CG.

Defects	% SG	% CG	*p* value
Demarcated opacities white/cream	24.6%	42.1%	0.0096*
Demarcated opacities yellow/brown	3.3%	0%	0.1229
Diffuse‐lines	11.5%	5,3%	0.0044*
Diffuse patchy	29.5%	5.3%	0.00001*
Diffuse‐confluent	21.3%	42.1%	0.0275*
Confluent/patchy + staining + loss of enamel	6.6%	5.3%	0.0562
Missing enamel	1.6%	0%	0.3481
Pits	1.6%	0%	0.3481

Figures S1, S2, and S3 show examples of the DDE observed, respectively, demarcated opacities (white/cream‐colored), diffuse patchy opacities, and diffuse‐confluent enamel defects.

When observing the differences in DDE depending on the type of treatment received by deciduous teeth with endodontic abscess, 47/107 deciduous molars were treated with ET, while 60/107 were E. In the corresponding premolars, DDE were found in 33/47 (70.2%) of the ET group and 28/60 (46.7%) of the E group. The prevalence of DDE was significantly higher in premolars following ET than in those following E, with an OR of 2.69 (95% CI 1.20–6.03 *p* = 0.016).

Figures S4, S5, and S6 show, respectively, a case where the deciduous molar was extracted, a case where the deciduous molar underwent root canal treatment, and an example of deciduous tooth diagnosed with an apical abscess.

When observing the impact of anatomical differences, maxillary premolars had a prevalence of DDE of 73% (46/63), while mandibular premolars had a prevalence of DDE of 34.1% (15/44). This difference was statistically significant, with an OR of 5.23 (95% CI 2.27–12.06; *p* = 0.00008). This confirms that maxillary premolars are significantly more likely to present DDE compared to mandibular premolars.

Table 2 shows the demographic and clinical characteristics of SG and CG.

**Table 2:** Demographic and clinical characteristics of SG and CG.

Variable	Study group (SG) (*n* = 107)	Control group (CG) (*n* = 107)	*p* value^a^
Age (years)	13.07 ± 3.7	13.07 ± 3.7	1.000
Sex			
Male	52 (48.6%)	52 (48.6%)	1.000
Female	55 (51.4%)	55 (51.4%)	
DDE present	61/107 premolars (57.0%)	19/107 premolars (17.8%)	< 0.0001*
Treatment type of deciduous molar		N/A	—
Extraction (E)	60/107 (56.1%)	—	—
Endodontic (ET)	47/107 (43.9%)	—	—
DDE in ET group	33/47 (70.2%)	—	—
DDE in E group	28/60 (46.7%)	—	—
Tooth location			—
Maxillary premolars	63	63	—
Mandibular premolars	44	44	—

^a^ Fisher's exact test or *t*‐test where appropriate; *p*‐values are reported for age and sex only due to the matching design.

* Statistically significant *p*‐values.

## 4 Discussion

The impact of endodontic abscess on permanent tooth formation remains largely unstudied. Some articles describe an association between periradicular inflammation and infection on deciduous teeth and alteration in eruption and/or development of the correspondent permanent teeth (Mariela Rodriguez Cordeiro and Jose de Carvalho Rocha 2005; Patil et al. 2023; Li et al. 2023); however, to the best of our knowledge, this is the first study examining the effect of chronic abscesses on deciduous teeth, on the corresponding permanent teeth. In fact, recent systematic reviews suggested an association between DDE and dental caries (Gevert et al. 2024; Costa et al. 2017); however, authors mainly focused on dental caries as consequences of DDE, rather than studying the cause of DDE.

In the present study, a high prevalence of DDE has been observed in premolars whose predecessors suffered from endodontic abscesses, confirming our hypothesis. Some studies reported an impact of dental caries lesion on the occurrence of demarcated opacities and hypoplasia in permanent teeth (Lo et al. 2003; Olczak‐Kowalczyk et al. 2023); the present study confirms and better describes these findings, by showing that chronic endodontic abscesses on deciduous molars are a significant risk factor (OR = 6.14) for the development of enamel abnormalities on premolars. Possible explanations for these findings could be attributed to the proximity of the deciduous tooth to the permanent tooth germ, as demonstrated by scanning electron microscope studies (Da Silva et al. 2009).

The most common type of DDE observed in the SG was diffuse patchy opacities, accounting for 29.5% of cases. This was followed by white/cream delimited opacities (24.6%), diffuse‐confluent opacities (21.3%), and diffuse lines (11.5%). Less frequent were confluent/patchy opacities combined with staining and loss of enamel (6.6%), yellow/brown delimited opacities (3.3%), and missing enamel and hypoplasia (1.6%). Differences are observed compared to the CG, where the most prevalent abnormalities were white/cream delimited opacities and diffuse‐confluent opacities (42.1%), followed by confluent/patchy combined with staining and loss of enamel, as well as diffuse patchy opacities and diffuse lines, each observed in 5.3% of the premolars. These findings are in agreement with the most common DDE observed by Jälevik et al. (2018). According to Bauer, the increased prevalence of these defects is linked to the timing of abscess occurrence and correlates with the period of enamel maturation in the developing tooth (Bauer 1946).

The findings of this study indicate that when molars affected by endodontic abscesses are managed with conservative therapy, the likelihood of developing DDE in the underlying developing permanent premolars is higher than in those treated with E (OR = 2.69). This increased prevalence may be linked to the type of material used in the ET, which could potentially diffuse beyond the apex and disrupt the amelogenesis process in the developing tooth germ (Al‐Ostwani et al. 2016). Conversely, the study by Sousa et al. (2020) reports a greater frequency of DDE in premolars whose predecessors were extracted early due to dental necrosis, compared to those treated with ET. A possible explanation of this difference can be related to the treatment timing, as it can be hypothesized for E to be differed. Another possible explanation is traumatic damage to the enamel tissue of the underlying tooth during the surgical procedure (Sousa et al. 2020).

While our study shows a higher prevalence of DDE following endodontic treatment compared to extraction, we recommend that the decision between extraction and endodontic treatment should be made on a case‐by‐case basis, considering the severity and duration of the endodontic abscess, the developmental stage of the permanent successor, and the patient's overall dental health and compliance. Our findings highlight the importance of thorough preoperative assessment and careful consideration of treatment modalities to minimize potential impacts on developing permanent teeth and future orthodontic complications.

In line with the studies by Lo et al. (2003) and Van der Weijden et al. (2020), our findings confirmed and reinforced the finding that maxillary premolars are more affected by DDE compared to mandibular premolars (OR = 5.23). A possible explanation could be the lower density of the maxillary bone compared to the mandible, which allows for more spread of the inflammatory infiltrate (Truhlar et al. 1997).

The main limitation of the present study stands in its cross‐sectional nature, which precludes the ability to draw definite conclusions about causal relationships between chronic endodontic abscesses in primary molars and DDE in the corresponding permanent premolars. Future studies should adopt longitudinal designs to better assess this association. Further, the study did not account for the timing between the endodontic abscess and the treatment provided, which could have potentially played a role in the spread of inflammation and therefore in DDE development. Due to the retrospective nature of data collection, it was not possible to define a clear temporal reference point for each case. As a result, we were unable to calculate risk ratios or incidence rate ratios, which would have allowed for a stronger causal interpretation of the findings. In the absence of such temporal data, the odds ratio remained the most appropriate and feasible measure to assess the association between chronic endodontic abscesses in primary teeth and DDE in the corresponding permanent premolars. However, it is important to acknowledge that, in certain contexts, odds ratios may overestimate the strength of association compared to risk ratios, particularly when the outcome is common. Therefore, while our findings indicate a significant association, they do not establish a direct causal relationship. This limitation has been considered in our interpretation and should be addressed in future prospective and longitudinal studies.

Despite these limitations, the current study also has several strengths, including a large sample and the use of validated questionnaires, which enhance the reliability of the results.

Moreover, it reinforces the association between DDE and endodontic abscess and provides compelling data on the risk of DDE development.

Clinically, these findings highlight the importance of regular dental checkups and timely dental treatment, aiming to reduce the risk of endodontic abscess and subsequent DDE development.

## 5 Conclusion

Within the limitations of the current study, our findings revealed that the formation of chronic endodontic abscesses in primary molars is a significant risk factor for DDE in the corresponding underlying premolars. The type of treatment performed on the affected primary molar influences the prevalence of DDE in the underlying permanent teeth. Specifically, premolars whose predecessors underwent ET show a higher incidence of DDE compared to those that were extracted. The most frequently observed enamel anomalies in permanent premolars whose predecessors presented chronic endodontic abscesses were diffuse patches (29.5%), demarcated white/cream opacities (24.6%), and diffuse‐confluent opacities (21.3%). Additionally, maxillary premolars were found to be more prone to DDE than mandibular premolars.

## Author Contributions

Federica Guglielmi contributed to conception and design and critically revised the manuscript. Angela Malatesta contributed to data acquisition and drafted the manuscript. Anna Alessandri‐Bonetti contributed to data acquisition and critically revised the manuscript. Patrizia Gallenzi contributed to conception and design and critically revised the manuscript. All authors gave their final approval and agree to be accountable for all aspects of the work.

## Ethics Statement

The study protocol was submitted for evaluation by the Ethics Committee of the Fondazione Policlinico A. Gemelli IRCCS and was accepted by Prot. No. 0030896/23 on October 31, 2023. Only patients who had a written consent from parents/caregivers were included in the study.

## Conflicts of Interest

The authors declare no conflicts of interest.

## Supporting information


**Figure 1:** Demarcated opacities (whitecream‐colored).

**Figure 2:** Diffuse patchy opacities.

**Figure 3:** Diffuse‐confluent enamel defects.

**Figure 4:** A case where the deciduous molar was extracted.

**Figure 5:** A case where the deciduous molar underwent root canal treatment.

**Figure 6:** A deciduous tooth diagnosed with an apical abscess.

## Data Availability

The data that support the findings of this study are available on request from the corresponding author. The data are not publicly available due to privacy or ethical restrictions.
