# Supplementary figures and images for: Developmental Defect of Enamel in Permanent Teeth Associated With Chronic Endodontic Abscess in Deciduous Teeth: A Retrospective Study
Source: Clin Exp Dent Res. 2025 Jul 23;11(4):e70185. doi: 10.1002/cre2.70185 (PMC12285902; doi:10.1002/cre2.70185)

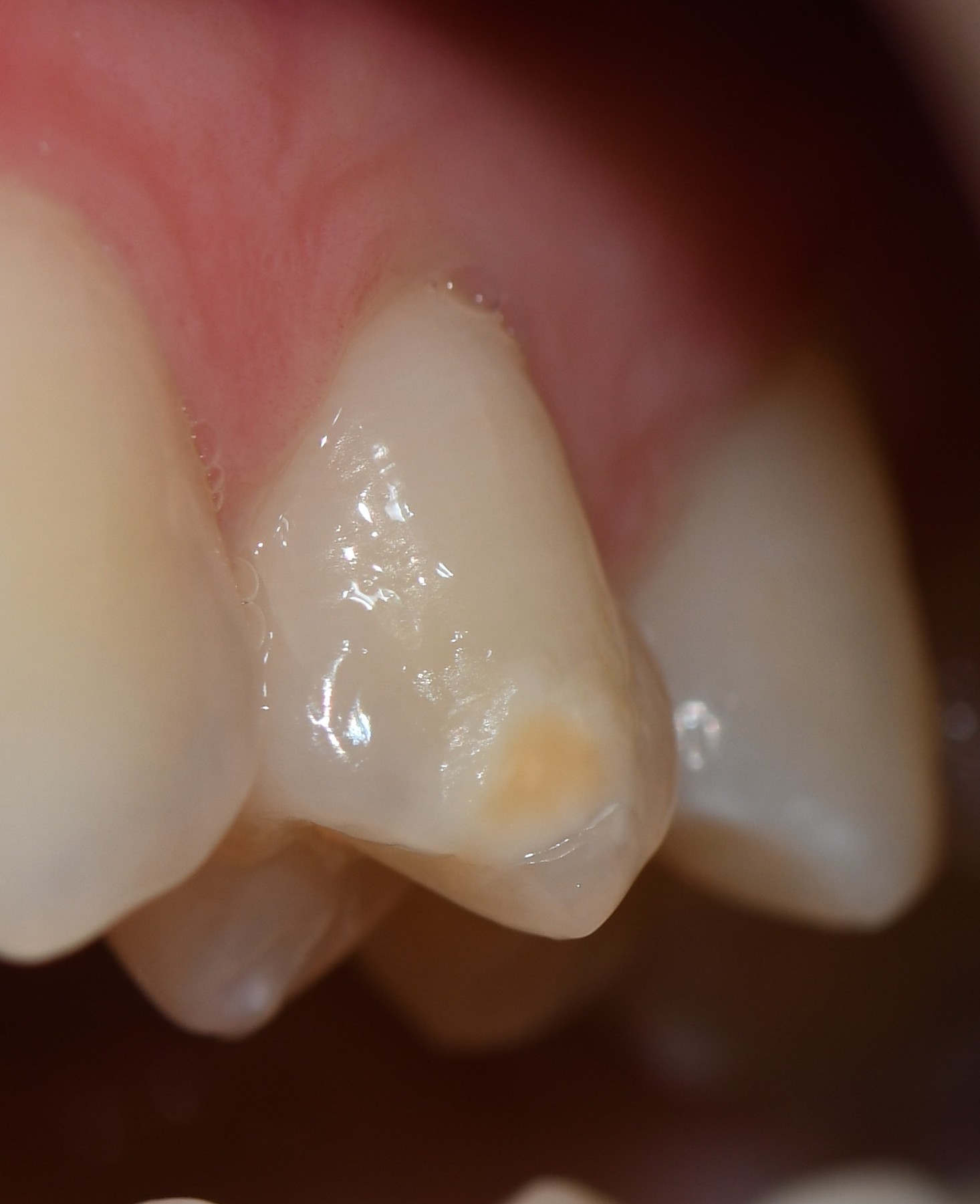

Supplement: Supplementary file 1 — Figure 1: Demarcated opacities (whitecream‐colored). [file CRE2-11-e70185-s003.JPG]

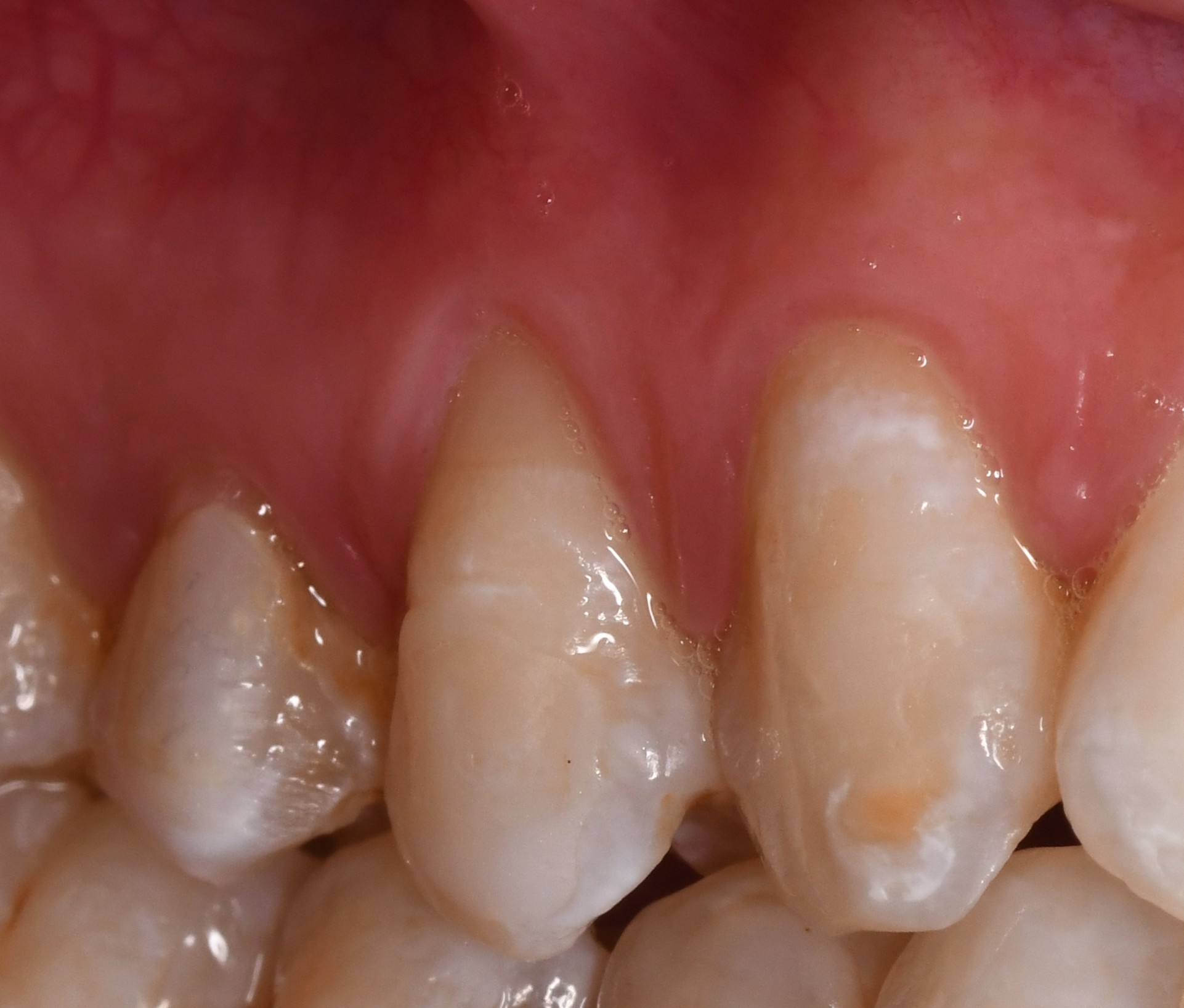

Supplement: Supplementary file 2 — Figure 2: Diffuse patchy opacities. [file CRE2-11-e70185-s005.JPG]

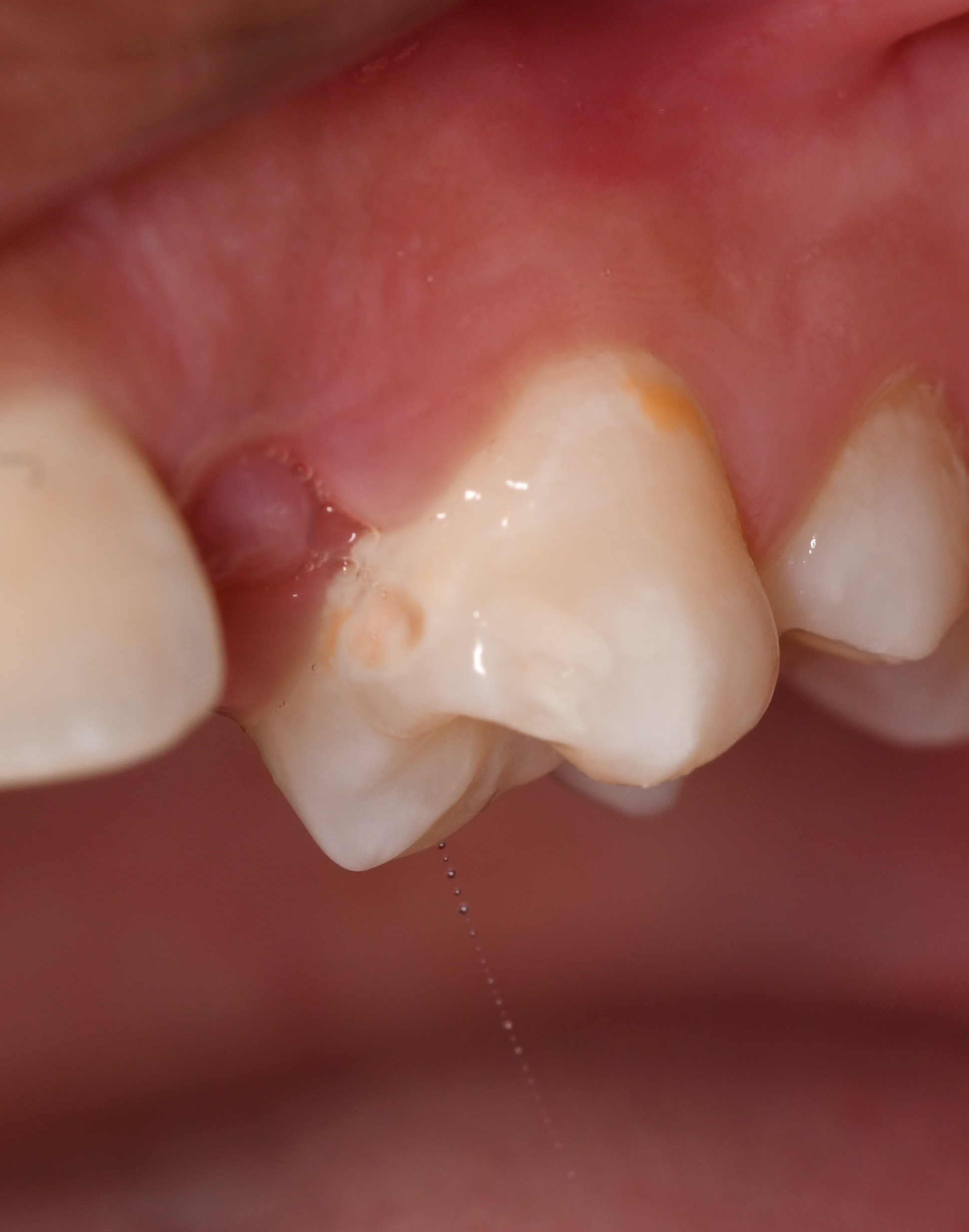

Supplement: Supplementary file 3 — Figure 3: Diffuse‐confluent enamel defects. [file CRE2-11-e70185-s006.JPG]

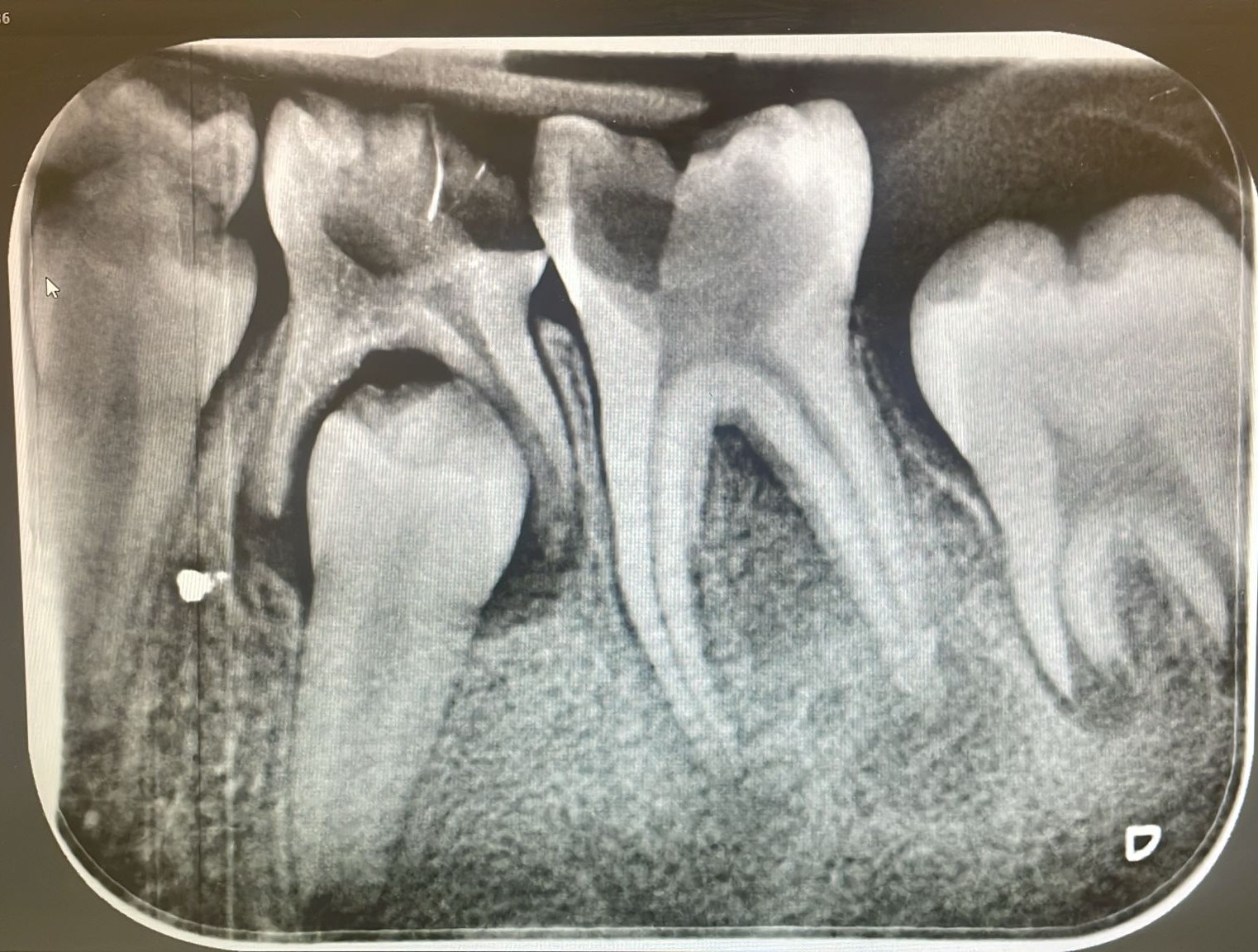

Supplement: Supplementary file 4 — Figure 4: A case where the deciduous molar was extracted. [file CRE2-11-e70185-s001.jpeg]

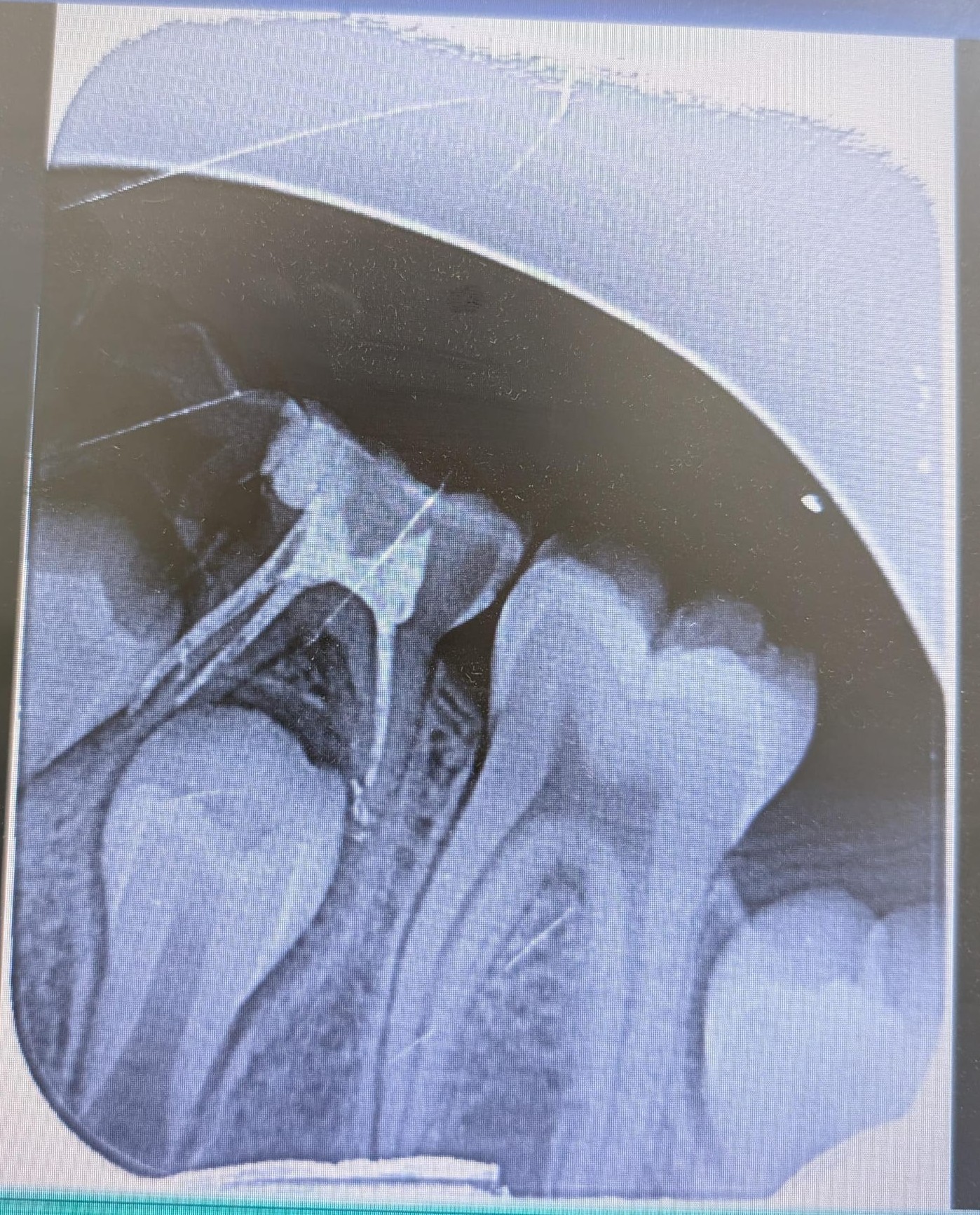

Supplement: Supplementary file 5 — Figure 5: A case where the deciduous molar underwent root canal treatment. [file CRE2-11-e70185-s004.jpeg]

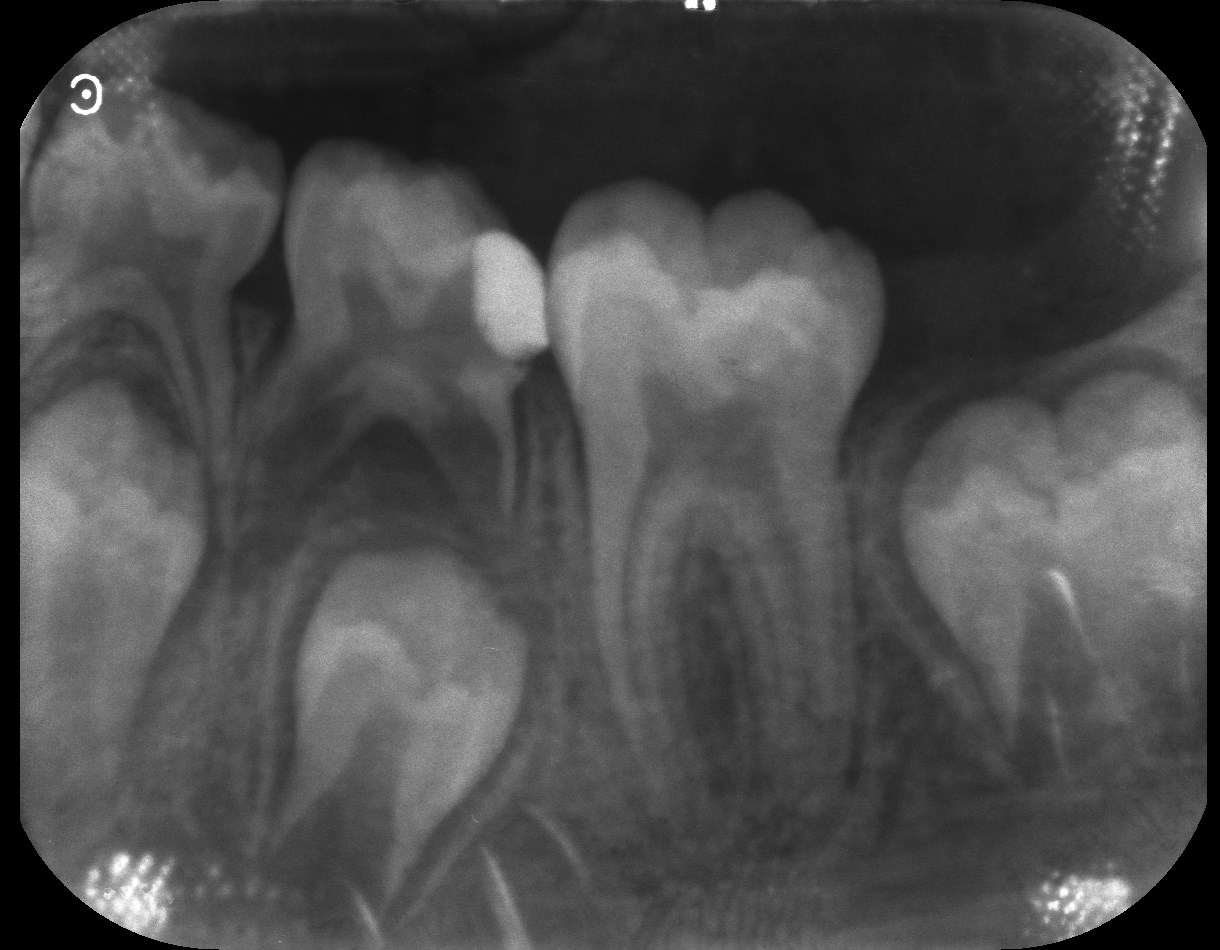

Supplement: Supplementary file 6 — Figure 6: A deciduous tooth diagnosed with an apical abscess. [file CRE2-11-e70185-s002.jpeg]
